# Designing a co‐productive study to overcome known methodological challenges in organ donation research with bereaved family members

**DOI:** 10.1111/hex.12894

**Published:** 2019-05-06

**Authors:** Jane Noyes, Leah Mclaughlin, Karen Morgan, Abigail Roberts, Michael Stephens, Janette Bourne, Michael Houlston, Jessica Houlston, Sarah Thomas, Revd Gethin Rhys, Bethan Moss, Sue Duncalf, Dawn Lee, Rebecca Curtis, Susanna Madden, Phillip Walton

**Affiliations:** ^1^ School of Social Sciences Bangor University Bangor UK; ^2^ Major Health Conditions Policy Team, Directorate of Health Policy, Health and Social Services Group Welsh Government Cardiff UK; ^3^ NHS Blood and Transplant, North West Regional Office Liverpool UK; ^4^ Department of Nephrology and Transplantation Cardiff and Vale University Health Board, University Hospital of Wales Cardiff UK; ^5^ Wales Director of Services ‐ Cruse Bereavement Care Cymru Caerphilly UK; ^6^ Parents of the Youngest Organ Donor in Wales and Lobbyists for Organ Donation Wales UK; ^7^ The Centre of sign‐Sight‐Sound Conwy UK; ^8^ Churches Together in Wales National Assembly Policy Officer Cardiff UK; ^9^ NHS Blood and Transplant, South Wales, South West and South Central Cardiff UK; ^10^ Statistics and Clinical Studies NHS Blood and Transplant Stoke Gifford UK

**Keywords:** bereavement, co‐production, ethics, evaluation, health services research, organ donation

## Abstract

**Background:**

Co‐production of research into public health services has yet to demonstrate tangible benefits. Few studies have reported the impact of co‐production on research outcomes. The previous studies of organ donation have identified challenges in engaging with public organizations responsible, gaining ethical approval for sensitive studies with the recently bereaved and difficulty in recruiting bereaved family members who were approached about organ donation.

**Objective:**

To address these challenges, we designed the first large co‐productive observational study to evaluate implementation of a new system of organ donation in Wales. This paper outlines the co‐productive strategies that were designed to overcome known methodological challenges and reports what impact they had on resolving these challenges.

**Design:**

Two‐year co‐produced study with multiple stakeholders with the specific intention of maximizing engagement with the National Health Service arm in Wales responsible for organ donation, and recruitment of bereaved family members whose perspectives are essential but commonly absent from studies.

**Setting and participants:**

NHS Blood and Transplant, Welsh Government and multiple patient and public representatives who served as co‐productive partners with the research team.

**Results:**

Co‐productive strategies enabled a smooth passage through four different ethics processes within the 10‐week time frame, family member recruitment targets to be surpassed, sharing of routinely collected data on 100% of potential organ donor cases and development of further research capacity and capability in a critically under researched area.

**Discussion and conclusion:**

Although expensive and time consuming, co‐production was effective and added value to research processes and study outcomes.

## 1 INTRODUCTION

This paper focuses on the impact of co‐production on research processes and outcomes. Co‐production, co‐creation, participation and involvement are some of the terms used interchangeably to describe research that includes non‐academics. When co‐production is used as a research method, it means that non‐academics—those citizens not employed by a university to undertake research—are invited to collaborate equally in all aspects such as the design, recruitment and dissemination of the research.^1^ Co‐production does not however mean that the roles of the academic and non‐academics dissolve or switch.^2^, ^3^

There is often a tacit assumption that co‐production will inevitably produce better research. The evidence for the benefits of co‐production to the quality of research processes and outcomes is still scant. A recent systematic review of 122 articles and books that included co‐production showed that only a handful of studies focused on the outcomes of co‐production and what co‐production actually achieved.^1^ To date, research has mostly focussed on the impact of patient and public involvement in trials. In a recent systematic review of 26 trials, patient and public involvement modestly but significantly increased participant enrolment.^4^

### 1.1 Known methodological challenges in organ donation research

Changes to the management of the organ donation system have occurred in many countries.^5^ The impacts of such changes have typically not been explored through qualitative research with bereaved family members. The specific methodological challenges are outlined in Table 1.

**Table 1:** Methodological challenges, co‐production strategies, targets by which success was measured and outcomes of co‐production

Methodological challenge	Co‐productive strategies to address challenge	Target by which success was measured.	Outcomes of co‐production and what co‐production achieved
Lack of organizational commitment to support sensitive organ donation research.^6^	Welsh Government and NHSBT identified as key partners, along with key patient and public representatives Support gained from Head of NHSBT Operations and Welsh Government Key professional and lay co‐applicants identified to jointly design the study and funding application Research Officer joined monthly NHSBT team meetings NHSBT staff invited to join research team meetings Every staff member/ patient and public representative presented with a study mug Joint all day meetings with co‐partners to design data collection tools and processes Residential 2 d interim findings event for all co‐partners Monthly newsletter Twitter feed Podcasts Staff/patient and public training and professional development events Partner involvement in data interpretation SNOD and managers recruited for interviews and focus groups as participants Shared findings and learning with Welsh Government End of study celebration event with partners Joint presentation of findings with partners to different audiences	All 23 SNODs and managers engaged in bereaved family member recruitment and recruitment targets met	All 23 SNODs and managers engaged in research process. Fifty patient and public representatives actively contributed to the study
		Engagement with sufficient patient and public organizations to co‐produce the study	Bereaved family member recruitment rates surpassed—see below and Figure 1
		Data sharing agreement agreed with NHSBT and Welsh Government and data shared on all potential organ donor cases	Data agreement signed by all parties and anonymized data shared on all potential prospective organ donor cases and up to 3 y retrospective data
		Attendance of co‐partners at meetings and events	All events over subscribed with wait list
		Minimum of 23 professional participants in interviews and focus groups	Nineteen professionals recruited (workforce had reduced and been reorganized)
		Organizational and patient and public partners present the findings	Partners took the lead on presenting findings jointly or alone at multiple events
Ethics committees are concerned about authorizing sensitive research with recently bereaved people when relatives are most vulnerable: the circumstances of a death where donation is a possibility are often unexpected and traumatic. Historical relationships (distant or close) and behaviour (supportive or antagonistic) may become apparent when family members come together in a confined space and under tragic circumstances. Memories of events may change over time if recruitment is delayed.^7^, ^8^, ^9^	Consultation with bereaved family members concerning ethical research approaches in this context Obtained letter of support from bereaved family members for the approaches taken Offers of attendance at the ethics committee by bereaved family members to support the research study Application of an evidence‐based ethical framework and distress protocol to inform interview practice with bereaved people Selection of research officers with specific empathetic skills Embedding bereavement support material from CRUSE Bereavement care in routine interview practice	Receipt of all ethics committee approvals within 10 wk of obtaining the funding letter	Ethics committee approvals obtained within 10 wk
		Bereaved family members willing to participate in interviews and finding it a positive experience	Bereaved family members were positive about being involved. CRUSE bereavement resources positively received
Difficulty recruiting bereaved family members resulting in insufficient participant numbers to adequately explore the research questions. Embedded culture of protecting the anonymity of donor families, preconceptions that participating in research was too distressing for potential donor families, difficulty engaging with the National Health Service (NHS) organ donation workforce (NHS Blood and Transplant NHSBT) and staff not following the processes set‐up for the recruitment of donor families to research.^6^	Worked with multiple people and public representatives to develop sensitive study processes and to interpret data Multiple recruitment strategies designed with SNODs and patient and public representatives SNODs identified as main recruiters due to their relationship with bereaved family members and communication skills Embedded participant recruitment into routine SNOD procedures	Recruitment of family members of a minimum of 50 potential organ donor cases	Eighty‐eight family members of 60 potential organ donor cases were included. SNODs recruited 93% of those family members interviewed
			Only 31/211 questionnaires (one data collection option) received from family members. Family members preferred a face‐to‐face interview

**Figure 1:**
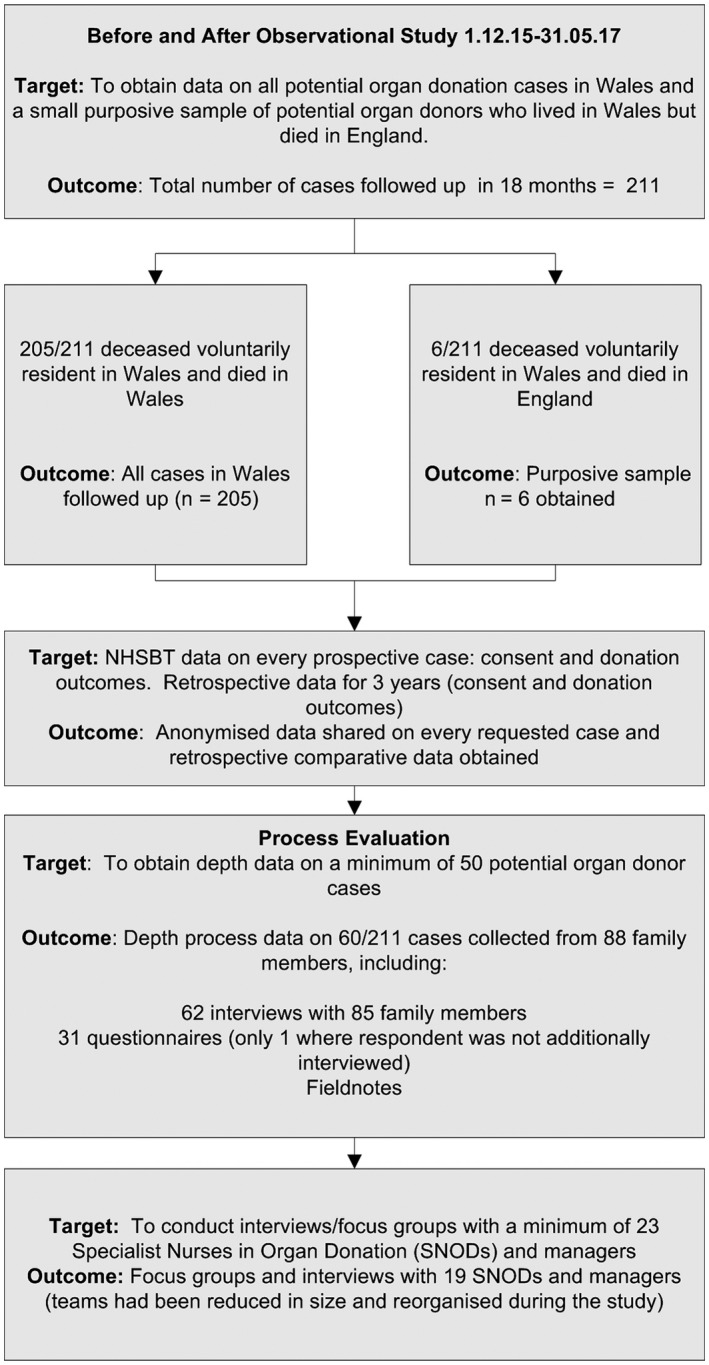
Study design and recruitment

### 1.2 Evaluation of the new soft opt‐out system in Wales

In 2015, Wales changed to a soft opt‐out system of organ donation. A comparison of the old and new system is shown in Table 2.

**Table 2:** Comparison of previous opt‐in and new soft opt‐out system

Decision type					
	Active	Passive	Family consent	Geographical reach	Role of family
Former opt‐in system	Register to opt‐in on the organ donor register Verbally tell a relative or friend you want or do not want to be a donor Write telling a relative or friend you want or do not want to be a donor Nominate a representative to make the decision for you (Nowhere to record this decision)	Do nothing and remain a non‐donor unless your relative gives consent to organ donation	Person under 18 lacks mental capacity	UK wide	To give consent for organ donation if their relative has actively opted in or to make a donation decision on behalf of their relative
New opt‐out system in Wales	Register to opt‐in on the organ donor register Verbally tell a relative or friend you want to be a donor Write telling a relative or friend you want or do not want to be a donor Register to opt‐out on the organ donor register Appoint a patient representative on the organ donor register to make the decision for you	Do nothing and remain as a donor (Deemed consent)	Person under 18 lacks mental capacity	Wales only Welsh citizens have to die in Wales for the soft opt‐out to apply. If they die in England, the opt‐in system applies	To support the donation decision of their relative made in life Clinicians however acknowledged that they would not pursue organ donation if the family member refused to support their relative's donation decision made in life

In brief, we co‐designed and delivered a 2 years before and after observational study with 18 months post‐implementation follow‐up to achieve four objectives:

- To determine the impact on consent rates;
- To evaluate implementation processes;
- To explore the views and experiences of family members who were approached to discuss organ donation; and
- To further build research capacity in NHSBT and Patient and Public Involvement.

Elsewhere, we report the protocol,^10^ the impact of the soft opt‐out on consent rates^11^ and findings from the process evaluation.^12^ The study design, recruitment targets and outcomes are shown in Figure 1 as context to evaluating the impact of co‐production on study processes and outcomes in this paper. We hypothesized that co‐production could be a way to address the methodological challenges that the previous organ donation studies have encountered and summarized in Table 1.

### 1.3 Aims of this paper

This paper reports the co‐productive strategies used in a study to explore the four objectives outlined above. We specifically focus on the outcomes of co‐production, what co‐production actually achieved, and whether known methodological challenges in organ donation research were resolved in this study.

## 2 CO‐PRODUCTIVE METHODS

See Box for how we conceptualized the key responsibilities of the academic partner in this study.

BOX 1 **Key responsibilities and assumptions of the academic partner in our co‐productive study based on Voorberg et al, Bell & Pahl, and INVOLVE.^1^, ^2^, ^3^**

1

- To maximise the most appropriate opportunities for co‐production to “happen.” Co‐production acknowledges the various expertise, skills, views and perspectives of the academic and non‐academics and creates ways for collective working. The assumption being that multiple and different perspectives will enhance or provide new information relating to the phenomena of interest and key research questions.
- To seek out the different and expert perspectives relevant to the research topic. Generic input might be suitable but more often there will be particular “expert non‐academics” that will need identifying and targeting to participate.
- To ensure timely, ongoing and multiple recruitment strategies. Non‐academics will likely have little knowledge of research processes in their own field. The academic needs to contextualize the research to multiple audiences and present this at the appropriate times.

Table 1 summarizes the co‐production strategies that are further described in the subsequent sections.

### 2.1 Co‐production strategies with NHSBT and Welsh Government to enhance engagement

The broad aim was to build new partnerships with those organizations who were key to designing and delivering the new system of organ donation in order to overcome problems with lack of collaboration in the previous studies. These organizations were identified as NHS Blood and Transplant (NHSBT), who were responsible for operational delivery and Welsh Government who drafted the legislation and funded its implementation. In Wales, Specialist Nurses in Organ Donation (SNODs) are employed and trained by NHSBT. The role and responsibilities of the SNOD are outlined in Table 3.

**Table 3:** The main responsibilities of the Specialist Nurse in Organ Donation at the time of the study

Main responsibilities of the Specialist Nurse in Organ Donation at the time of the study				
Consent‐related activity	Clinical activity	Theatre	Hospital development	Potential donor audits
Triage incoming referrals Attend the referrals Approach families for consent	Engage with all clinical activity following consent Provide support for family members and staff	Attend theatre and help coordinate the retrieval procedure	Engage with hospitals to drive referrals to ensure hospitals comply with transplant process Engage in education and practice development activities	Audit files of all people who die in the emergency department and intensive care unit below the age of 81

At the time of the study, there were two regional NHSBT teams covering Wales: the North West England team covering North Wales, and the South Wales team. The teams had a combined workforce of 32 SNODs, six team managers, two practice development specialists, two regional managers and administration staff. From this pool, one former senior NHSBT manager who was subsequently seconded to Welsh Government (Morgan), a Regional Manager (Walton), a Specialist Nurse in Organ Donation (Roberts) and a transplant Surgeon (Stephens) were contacted and became co‐applicants on the original grant application and were involved in all stages from question development and research design to the practical implementation of the study. The Head of Operations, Organ Donation and Transplantation at NHSBT (Wellington) wrote a letter supporting the study.

Key Welsh Government staff responsible for the new organ donation policy (Vernon, Jones, Lewis) provided letters of support and access to internal Government documents and intelligence to support development of the grant application. Morgan provided ongoing continuity of communication with Welsh Government as the study progressed.

### 2.2 Co‐production strategies with patient and public representatives to facilitate engagement

The broad aim was to build a consortium of patient and public representatives to help overcome the ethical challenges of interviewing recently bereaved people and known difficulties in recruitment. As the study primarily involved interviewing recently bereaved people, we partnered with CRUSE Cymru (the major not for profit bereavement support organization in Wales), who provided bereavement support literature for research officers to share with family members. CRUSE Cymru also provides a free bereavement support service. In return, the research team agreed to host a fund raiser for CRUSE midway through the study. The Director (Bourne) joined the study steering group.

The change in law which introduced the soft opt‐out meant that every citizen in Wales was a potential organ donor unless they expressed otherwise. We needed to ensure that the study design and delivery had input from an all Wales perspective. High profile donor families in Wales (who had shared their stories publicly in the media) advised on the study set‐up and joined the study steering group. We also needed to capture the views and experiences of the Black, Asian, Minority and Ethnic (BAME) populations in Wales. The BAME population living in Wales is small and has lower organ donation rates than the Welsh population. To address this evidence gap, we focussed our public engagement on organizations and groups specifically representative of these communities. We used Internet searches, email invitations, follow‐up phone calls and then face‐to‐face meetings to recruit a range of representatives from under‐represented groups in Wales.

### 2.3 Other co‐productive strategies to maintain organizational and patient and public commitment and enhance research processes

#### 2.3.1 Co‐production strategies to develop data collection procedures for each potential organ donor case

The aim of the co‐productive approach was to engage with SNODs for the purposes of collecting data. In the UK, SNODs are employed by NHSBT and deployed to meet family members when a potential organ donor is identified by hospital consultants. They are tasked with recording data on every case (Table 2). The study was co‐designed to share anonymized routinely collected NHSBT data via a data sharing agreement and for SNODs to prospectively collect additional data on each case. The target was to collect a minimum data set on every potential organ donor for 18 months commencing 1 December 2015. For the purposes of this study, a potential organ donor was defined as a patient who is eligible for organ donation and whose family is approached for a formal organ donation discussion. The research team funded and facilitated a one‐day co‐productive meeting with each NHSBT team to support development of the data collection tools (Data S1). All SNODs worked on iPads, so we designed their anonymized data collection tool to be sent from their iPads to a central specially set up NHSBT email account and forwarded to the research team in batches by a nominated NHSBT administrator. This initial meeting also involved an element of research training for SNODs. SNODs were also presented with a study mug.

#### 2.3.2 Co‐production and interpretation of findings

The aim was to co‐produce a shared understanding of the findings and what they meant with all co‐productive partners. A 2‐day residential meeting at 12 months and an end of study event were convened to discuss and interpret findings. Early findings at 12 months were shared with NHSBT and Welsh Government to act on. See Data S2.

#### 2.3.3 Co‐production and ongoing study communication

The aim was to use co‐production to maximize study communication. For ongoing communication, research officer (McLaughlin) joined relevant elements of monthly NHSBT team meetings with the respective teams for over two years and the study became a regular agenda item. She also attended retraining events for SNODs hosted by NHSBT, and NHSBT staff were invited to join virtual weekly core research team meetings. Additional members of NHSBT staff served on the advisory group, which met twice. Additional strategies included fortnightly newsletters produced by the research team containing updates about recruitment and data collected, events and news items fed back to an email group of over 100 people including SNODs and patient and public representatives (Data S3). The research team filmed and released a podcast video with a demonstration of filling out consent to contact forms and created a Twitter and study website. Extensive research team member time was allocated to being available to attend NHSBT events, conferences, team meetings and informal catch‐ups with SNODs wherever possible in their workplaces (Data S4).

#### 2.3.4 Co‐production and dissemination

The aim was for co‐productive partners to present findings to their communities and to co‐present findings to academic, clinical and policy communities. The researchers provided support and training for this activity. The research team also facilitated an end of study training event with SNODs and managers with a trained crisis negotiator, which picked up on selected study findings that needed addressing.

### 2.4 Co‐production strategies to gain timely ethics committee approval

The broad aim was to use a co‐productive approach to ensure the timely receipt of ethical approval to commence the study. From receiving the funding award letter, the research team had 10 weeks before the soft opt‐out was implemented on 1 December 2015 to obtain the relevant permissions from an NHS ethics committee, NHSBT Research, Innovation and Technology Group (RINTAG) and NHSBT Research and Development approval to commence the study. The Chief Investigator (Noyes) met in person and was advised by NHSBT Organ Donation and Transplant Research Manager (Brailsford), who helped navigate NHSBT internal processes and procedures, commented on draft applications and liaised with the various committees.

The research team consulted with family members who had previously been approached about organ donation to ascertain their views on conducting research with recently bereaved people in similar circumstances. Michael and Jessica Houlston (parents of an organ donor) subsequently wrote a letter to the NHS ethics committee outlining that they supported the proposed research design, family members wanted to be asked their views and opinions as they had a lot to say that could help further improve processes, and family members should be given the same privileges as anybody else to participate in a research study. Other family members also offered to attend the NHS ethics committee in person with the Chief Investigator.

The research team adopted an evidence‐based ethical framework developed by UK‐based researchers for undertaking interviews with the recently bereaved people (Data S5). The framework included three key overall areas for consideration with associated recommendations, including participant identification and recruitment, the research interview and post‐interview follow‐up care.^13^ The framework was adapted to accommodate undertaking interviews within the first three months after bereavement. A distress protocol was also developed to show how researchers would respond if family members became very upset during interviews (Data S6).

### 2.5 Co‐production to recruit bereaved family members

The aim of the co‐productive approach with SNODS was to work out how to access and then recruit bereaved family members. The target was to ascertain detailed views and experiences from family members who represented a minimum number of 50 potential organ donor cases. We co‐designed multiple opportunities to recruit potential donor family members and developed more inclusive strategies to work closely with NHSBT to address any perceived barriers to recruiting complex cases, family member(s) who declined donation, and family member(s) who overrode their relative's organ donation decision. The research team hypothesized that family members may prefer an anonymous option to participate by returning a questionnaire. Patient and public representatives suggested that family members would likely prefer a face‐to‐face interview so we offered both options (questionnaire and interview) in English and Welsh. Family members could indicate on the consent to contact form when they wanted to be contacted by the researcher after the death of their relative.

#### 2.5.1 Recruitment strategies via SNODs

We hypothesized that the SNOD's judgement and specialist skills were best placed to introduce the family to the study at an appropriate time and in an appropriate way. Through co‐production, we engaged directly with the specialist skills of the SNODs. SNODs are highly trained specialist nurses who routinely manage very distressing situations. They have particular character traits which support this highly sensitive role such as empathy, patience, multi‐tasking, listening, trusting, confidence and management skills. We wanted to take advantage of these specialist skills and adapt them to support study recruitment. We planned to dovetail participant recruitment into the study with the care and well‐being of family members provided by SNODs. We wanted SNODs to ensure that the appropriate levels of care, support and understanding were in place before seeking consent to recruit bereaved family member(s). To achieve this, the research team aimed to build a relationship with every SNOD who would approach family member(s) who fitted the inclusion criteria (every family member over 16 where the deceased person was voluntarily resident in Wales and died in Wales or England) over the 18‐month data collection window (01.12.15‐31.05.17). We facilitated opportunities to immerse the SNODs wherever possible in the study and to design ways to “normalize” the study into their everyday practice. We felt that by creating an environment of shared learning and opportunities for feedback and open discussion, the SNODs would find genuine value in the research and take a real ownership in terms of delivering the study aims. We jointly specified three clear pathways to recruit family members which mapped directly onto the SNODs practice at the time of the study: 1. at the end of “the approach” conversation, 2. during SNOD routine telephone follow‐up and 3. a postal follow‐up alongside a routine service evaluation form. These pathways were developed into an operating procedure, which included prompts for introducing the family member(s) to the study and shared with the SNODs. A3 colour posters were sent to their hospitals, and base locations to put on the wall as daily reminders of the study.

We needed to find ways to make the SNODs feel comfortable working with an (as yet) unknown research team, learn in‐depth detail about their practices in approaching families and tease out any concerns or issues they might have with asking a family to participate in a research study. We hosted a full day training session with the North West England and South Wales NHSBT SNOD teams. Those who missed the day(s) due to work commitments or illness were followed up with a mini session with a researcher at a convenient date.

Welsh/English study information packs for families were sent to hospitals and kept centrally in local NHBT offices to replenish. The central NHSBT administration hub was responsible for adding a pack to the SNODs “donor files,” a sticker with a Welsh flag, and study mark was also added to the front of the donor files as a reminder.

When a family member agreed to participate via the SNOD following their approach conversation, SNODs supported family member(s) to fill out a simple consent to contact form and then sent the form back to the research team via a prepaid envelope. The research team processed the contact details and followed up independently with family member(s). The consent to contact forms were logged, and researchers followed up with a telephone call at least four weeks later or at a time specified by the family on the form. This timeframe for an introductory call gave enough time for the funeral to pass but was close enough for the organ donation conversation to still be in the immediate memory of family member(s). Several attempts were to be made to contact family member(s) before leaving a telephone message. The telephone message invited a callback. If the research team did not receive a callback, we finally sent a postal follow‐up with an invitation to get in touch. The initial phone call was designed to introduce the family member to the study in more detail and seek agreement for a formal interview.

#### 2.5.2 Other recruitment strategies

Posters concerning the study were located at strategic places in hospitals where family members were likely to congregate. We asked family members who agreed to participate in an interview to pass on information about the study to others involved in the donation decision. A website and social media page linked to the study Twitter account was signposted in the study information booklet and consent to contact forms. We paid for two advertisements in the Daily Mail (the UKs' most read newspaper) on the Easter 2016 bank holiday and the August bank holiday 2016 as well as two local bilingual (Welsh/English) press releases with invitations for anybody who had been involved in an organ donation conversation to get in touch with the research team.

#### 2.5.3 Co‐production strategies for interviewing recently bereaved family members

The broad aim was to co‐produce a person‐focused and sensitive approach to interviewing recently bereaved people. Patient and public representatives supported development of the study materials that the research team were planning to give to family member(s) including the content and design of the cover letter and study information booklet and questions asked. SNODs also had a key role shaping what the researchers asked the family member(s) and how we asked it (interview topic guide and family questionnaire). Family members were asked to complete a questionnaire and or participate in an interview (face to face or via their chosen social media).

Research officers were selected for their empathetic skills and conducted all interviews with family members in Welsh or English. Interviews were recorded, transcribed verbatim and coded. Weekly research team meetings included time for reflection on the obvious sensitive content, and very often, tragic circumstances of the death were necessary for safeguarding the well‐being of the research team. Research team members had the option to contact CRUSE to reflect on any of the stories and emotions around bereavement that they were encountering on a regular basis, and to attend SNOD meetings where their experiences and strategies for engaging with bereaved families were discussed.

## 3 RESULTS

Table 1 summarizes the impact of co‐productive strategies on research processes and outcomes, which are described in more detail in the following sections.

### 3.1 Initial organizational and patient and public commitment

The co‐productive approach was successful. All 23 SNODs and managers in Wales and 50 patient and public representatives actively contributed to various research activities and processes to set up and deliver the study. A data sharing agreement was signed by all parties. All co‐productive events and activities to develop research processes were oversubscribed with a wait list.

Over 50 people representing their family or an organization were recruited. Organizations included Race Equality First, Women Connect First, Centre of Sign‐Sight‐Sound, Llanelli Multicultural Network, Churches Together in Wales, Donor and transplant families, Big Lottery and CRUSE bereavement care. A key part of their participation relied on the willingness of research team members to engage with them and their activities. The research officer (McLaughlin) participated in for example: chair yoga for Muslim women, dinner club, the “Advocacy for Elderly Ethnic Minority People (MEEA)” end of project conference, and local talks against discrimination and forced marriage. We were able then to recruit appropriate representatives to feedback into the study design and attend study‐related events. This engagement supported our responsibility to reflect back the perspectives of the BAME population in Wales.

### 3.2 Obtaining ethical approval

The co‐productive approach was successful, all approvals were received within 10 weeks, and data collection commenced as planned on 1 December 2015 to coincide with the switch to the soft opt‐out system. The protocol was approved on 23/10/15 by NHSBT Research, Innovation and Technology Advisory Group (RINTAG). This approval included agreement to share anonymized NHSBT data. The study was approved by the Wales Research Ethics Committee 5 (NHS research ethics committee) with very minor requested changes approved by the Chair (IRAS number 190066; Rec Reference 15/WA/0414 on 25/11/2015) and the NHSBT Research and Development Committee (NHSBT ID: AP‐15‐02 on 24/11/2015).

### 3.3 Recruitment outcomes

The co‐productive approach to recruitment was successful. Using shared anonymized NHSBT routinely collected data, all 205 potential organ donor cases in Wales were tracked from 1 December 2015 for 18 months, thereby achieving the target of obtaining data on 100% of cases. In addition, 6/38 potential Welsh organ donor cases who died in English hospitals were purposively sampled and followed up (making 211 cases in total). Family member recruitment targets to obtain additional depth data on a minimum of 50 cases were exceeded: 88 family members of 60/211 cases were included. SNODs recruited 93% of those family members interviewed. Consent to contact forms were received from 50% (93/186) of cases who were approached by a SNOD. SNODs also sometimes forgot to offer family member(s) an opportunity to participate in the study and needed reminding. Although we obtained additional depth qualitative data on 60/93 cases, there was insufficient time to include the remaining 33 cases, some of whom were lost to follow‐up or were at a stage of their bereavement where they were still not ready to participate in an interview, but would have liked to. We conducted depth interviews with 85 family member(s) and undertook 62 interviews: face to face (58) and phone (4). All but two of the face‐to‐face interviews were in a family member's home apart from (1) in a hotel lobby and (2) in a family member's workplace. We recruited no one in response to 2 expensive adverts placed in the Daily Mail and local papers. Only 31 questionnaires were received from family members, 28 of whom also participated in an interview. The successful recruitment rates are summarized in Figure 1.

The overall sample of family members was diverse and included a range of people who both supported and did not support the organ donation decision of their relative. The overall number of potential BAME organ donors was as anticipated very small. For example, there were only three approaches made concerning BAME potential organ donors from April 2016 to March 2017. We did however interview a couple of participants who were from BAME families and engaged with a wide variety of BAME groups to ensure that BAME perspectives helped shape the research.

Nineteen SNODs and managers provided depth data on their views and experiences of implementation in interviews and two focus groups, which matched the recruitment target.

### 3.4 Ongoing organizational commitment

#### 3.4.1 Interpretation of study findings

The co‐productive approach was successful, and multiple and different perspectives were obtained at the 2‐day residential event attended by over 80 people. Researcher interpretations were verified and in many cases amended or modified by key stakeholders.

#### 3.4.2 Building research capacity and disseminating findings

The study was successful at building research capacity, and co‐productive partners have presented findings. For example, Connect First co‐presented with the research team at the Annual Involving People Conference 2016 and Thomas (Community Support Manager) from Centre of Sign‐Sight‐Sound in Flintshire co‐presented at the Innovating Research Ideas in Kidney Health and Social Care Conference 2018. Bourne (CRUSE) presented at the final joint study event with all partners (Data S7). Walton (NHSBT) and Stephens (NHS Wales) have presented study findings at clinical meetings. Patient and Public representatives (for example Rhys) have provided detailed feedback on a new research application on organ donation involving existing partners. Moss has begun her own research project. The research team are working with Duncalf and Lee from the North West England team on an evaluation of a new “Specialist Requester” role.

## 4 DISCUSSION

To our knowledge, this is the first co‐productive observational study undertaken with family members to explore the impacts and outcomes of implementing a different system of organ donation. The study was awarded a national prize for the co‐production element. The study provides new insights and understanding about implementation of a soft opt‐out system of organ donation from the perspective of family members and professionals (See Box for summary of empirical study results).^11^, ^12^

BOX 2 **Brief summary of empirical results from the before and after study and embedded process evaluation. 11, 12**

1

**Table:** 

The big picture concerning consent rates	Organ donation consent rates improved to an overall rate of 61% in Wales. The difference was statistically significant, but could not be attributed to the new soft opt‐out system as consent rates had also similarly improved in England
The big picture concerning implementation	Specialist Nurses in Organ Donation generally felt that implementation went well. Examples of good practice included the innovative retraining programme and responsive approach to addressing initial implementation issues Family members, almost without exception, valued the professionalism of Specialist Nurses in Organ Donation Six important implementation issues were identified that impacted on consent rates: gaps in the media campaign concerning the changed role of family members; working within a more complex organ donation system; not being able to change professional behaviours; not being able to obtain the required standard of evidence for family members to overturn a decision, but the decision was overturned anyway; having to work with overly complex consent processes, and additional health systems issues such as the organ donation process taking too long so families withdrew consent

Co‐production worked well but it was an expensive research cost both in terms of resources (putting on events and activities) and researcher time spent working in collaboration with multiple partnerships. The Funder (Health and Care Research Wales) acknowledged and supported the role and value of co‐production in this context. Around £35 000 was allocated to cover the costs of patient and public engagement, residential meetings and embedded training opportunities for SNODs. This figure does not include researcher time and travel or the time of professional partners. We consider the additional costs of co‐producing the research to be good value for money. Creating an environment of shared learning and feedback at regular NHSBT team meetings, with Welsh Government representatives, and patient and public engagement opportunities, and a midpoint interim findings event built trust and rapport between the co‐productive partners. For example, Welsh Government commissioned a further media campaign to address the issues raised by family members about the lack of clarity concerning their role.

### 4.1 Research with family members as a potential positive opportunity

The general perception that bereaved family members are “too sad” to participate in research is misleading. Bereavement and grief are long‐term and normal processes, as are the range of feelings that often accompany a bereavement such as guilt, anger, depression and resentment. Whilst there is a need to be mindful and aware of the spectrum of circumstances that lead to deceased organ donation for families, it is equally important not to assume that any of these normal feelings render all bereaved people incapable of talking about them. It is however critically important to follow an individual approach and for researchers to respond to cues about participant readiness (or not) and ability (or not) to participate in research when recently bereaved. The ethical framework that was previously developed for family members provided a very useful tool to consider the needs of the bereaved person.^13^

Family member(s) were genuinely very interested in the topic and said that they found the experience of being interviewed soon after a bereavement a positive opportunity to share their story. Unlike Dyregory's study^14^ of bereavement with bereaved parents, none said that they found being interviewed especially painful or distressing. The main difference between Dyregory^12^ and the current study was that families were not being asked to primarily focus on their bereavement, and they fed back that sharing their experiences of organ donation was a positive experience if the information could be used to help others in similar situations.

There were tears and moments of obvious grief during interviews, but not always and not often. The distress protocol served as a useful framework for providing interviewees with the knowledge that they could stop at any time. Interviews were often interspersed with light‐hearted conversation. Some family member(s) were entirely matter of fact, talking through their experiences, and were especially keen to offer advice and suggestions for improvements to the organ donation process. All of the interviews offered something new to contribute to an overall understanding as to why consent rates improved and why family members still overrode their family member's donation decision.

Partnering with bereavement counselling services (CRUSE Bereavement Care Cymru) supported the conduct of the interviews with family members by differentiating the research and the research officers from a professional counselling service, as well as helping the research officers to disengage from family member(s) by signposting to additional bereavement support networks. Overall, we found that the information provided by CRUSE was particularly welcomed as family members appeared to lack access to bereavement support. Sharing the researcher's experiences of identifying the unmet bereavement care needs of those interviewed with study partners had the benefit of developing potential partnerships with CRUSE and NHSBT, as well as SNODs taking on local befriending projects in local communities in Wales. The research team hosted a Bring and Buy sale at the University, which raised £500 (Data S8). A cheque was presented to the Director (Bourne) at a 2‐day residential meeting with partners.

Of particular note, recruitment for interviews was far more successful than completion of anonymous questionnaires. As researchers, we expected to receive far more family questionnaires that could be shared anonymously. Three times more interviews were undertaken than questionnaires received, thereby confirming the view of patient and public representatives that face‐to‐face interviews were preferred by participants.

### 4.2 Impact of the study on researchers

There were many stories and cases which were exceptionally tragic. These cases often involved a suicide and/or the death of a young person. Undertaking face‐to‐face interviews with these family member(s) was at times emotionally challenging for interviewers. Whilst access to support and services were offered, none of the research team members took up the offer. They found that interviewing, listening and reviewing highly sensitive data were not sufficiently challenging to need additional support beyond that provided within the research team, and by working with SNODs who also had vast experience of working with bereaved people on a daily basis.

### 4.3 Ongoing challenges

Although the study was considered to be successful, there were however ongoing challenges. As in other studies, it was much easier to recruit family members who supported organ donation than those who did not. Additional emphasis on targeted recruitment by SNODs, and careful snowball sampling of family members, was needed to ensure the sample was sufficiently diverse and included those who did not support the organ donation decision of their relative. The strong partnership that developed with NHSBT enabled ongoing recruitment monitoring and joint meetings to respond when recruitment of particular types of family member participants was required.

Eighteen months of intense data collection was ample time to recruit the target sample of family members and ascertain sufficient data to evaluate short‐term outcomes. However, motivating and enthusing two NHSBT teams in a dynamic organization over this timeframe posed some specific challenges. There were major changes in administration (NHSBT centralized to one hub instead of regional administration teams) and practice (a new role, the Specialist Requester (a SNOD who focusses on gaining consent) was implemented in one team during the study). SNOD and manager turnover and sickness needed a flexible response by the research team to ensure continued engagement of new and returning staff with the study. There were noticeable dips in the return of data during especially busy periods, which in part likely resulted from structural changes. Providing flow charts and simple visual cues was found to be helpful reminders for SNODs to keep collecting and returning data, but face‐to‐face engagement and integration of researchers with NHSBT teams were vital for ensuring ongoing recruitment and data collection over an extended period of time.

## 5 CONCLUSIONS

This study contributed to developing co‐productive methodologies for recruiting recently bereaved people into research and interviewing on sensitive topics. Organ donation is a highly sensitive topic, and co‐production with multiple partners and patient and public engagement helped to demystify the research process and ensured that a study as sensitive as this had input from as broad a perspective as possible to maintain the high ethical standards demanded throughout.

With careful planning, the right partners and research staff, flexible options and choice for participants and the appropriate supports in place, recently bereaved family members can opt to be recruited to research studies and can be interviewed soon after their bereavement without additional stress, sadness or interference with the natural grieving process. Family member(s) although bereaved are able to make a choice as to whether they are ready to share their views, thoughts and experiences and are curious to learn more about the findings. Family member(s) prefer a face‐to‐face interview in their home than any other data collection method. An adequate size pool to recruit from is essential, but far more important is an engaged and enthusiastic recruiter (SNODs) to recruit sufficient participants with a diverse range of perspectives and experiences. Co‐production was a useful tool to build research capacity in a public health system and added value to the research study.

## CONFLICT OF INTEREST

There are no known competing interests.

## Data Availability

Study data are not publicly available. General NHSBT data are available at https://www.organdonation.nhs.uk/supporting-my-decision/statistics-about-organ-donation/.
