# Supplementary material for: Designing a co‐productive study to overcome known methodological challenges in organ donation research with bereaved family members
Source: Health Expect. 2019 May 6;22(4):824–35. doi: 10.1111/hex.12894 (PMC6737840; doi:10.1111/hex.12894)
Supplement: Supplementary file 1 [file HEX-22-824-s001.pdf]

Supplementary file 1. Setting up the study.

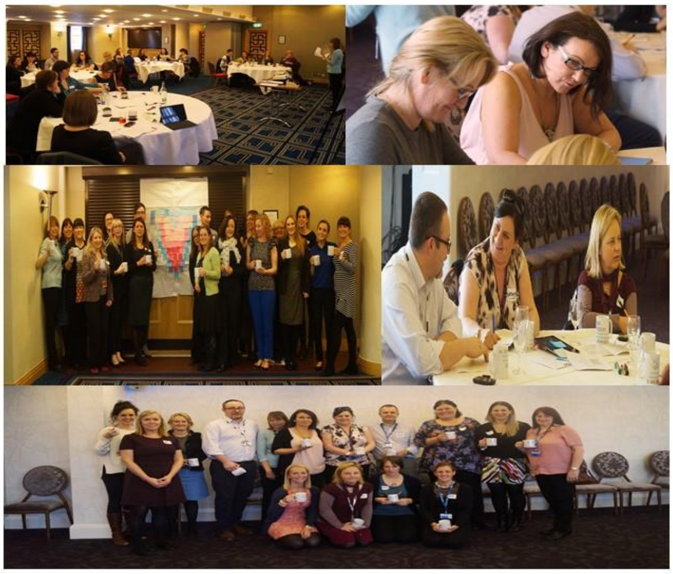

2 one day professional development days – with NHSBT SNODS, Management and support staff Oct/Nov 2015 Setting up the study.
