# Supplementary material for: Designing a co‐productive study to overcome known methodological challenges in organ donation research with bereaved family members
Source: Health Expect. 2019 May 6;22(4):824–35. doi: 10.1111/hex.12894 (PMC6737840; doi:10.1111/hex.12894)
Supplement: Supplementary file 2 [file HEX-22-824-s002.pdf]

Supplementary file 2. Interim findings event.

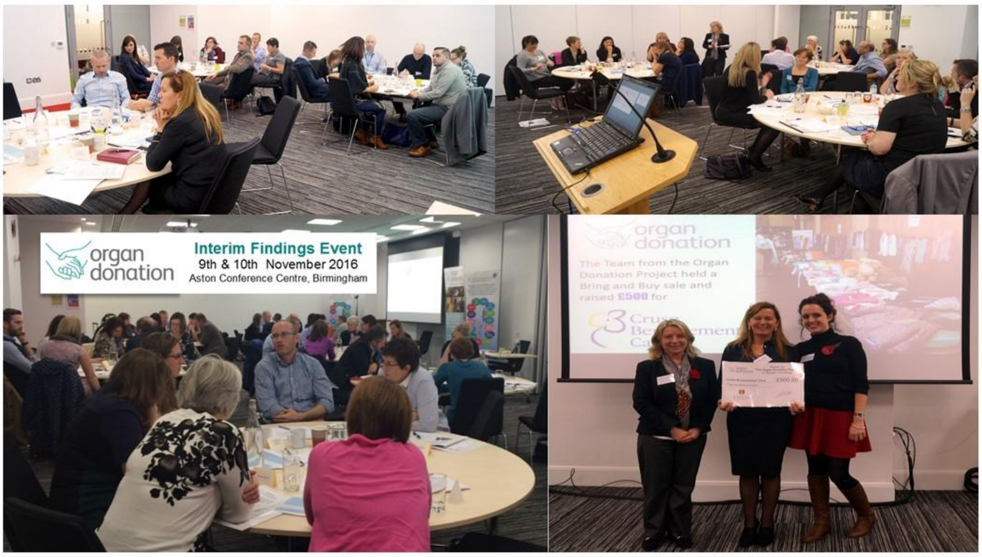

Two-day interim findings event. 50 key stakeholders invited to attend.
Fund raising check presented to Jeanette Bourne CRUSE Bereavement Care Cymru CEO.
