# Supplementary material for: Designing a co‐productive study to overcome known methodological challenges in organ donation research with bereaved family members
Source: Health Expect. 2019 May 6;22(4):824–35. doi: 10.1111/hex.12894 (PMC6737840; doi:10.1111/hex.12894)
Supplement: Supplementary file 3 [file HEX-22-824-s003.pdf]

Supplementary file 3. Sample Newsletter.

Organ Donation Study Newsletter

Welcome to our fortnightly newsletter providing you with updates and information about the study as it progresses.

Issue 21 **19th Sept 2016**

SNOD NEWS

It was "**Organ Donation Week**" last week in Cardiff. Many thanks to Guy, Charlotte and Sarah (Specialist Nurses in Organ Donation) for asking the Organ Donation Team to come along and support the event by hosting a stand in the Concourse in the Heath Hospital in Cardiff.

We all had a lovely morning and got to meet lots of passers-by and talked about Organ Donation in Wales.

The Organ Donation Research Team have been doing a lot of work over August to promote the study more broadly, this helps to promote organ donation in Wales and potentially to connect with more families who have had a bereavement and would like to speak to us. Promotion includes (posters in hospitals, adverts and press releases in papers, interviews on local radio and speaking at community events). However, none of this outreach activity compares to the engagement of the SNOD and their vital role in introducing families to the study. Once the family has disengaged from the SNOD the potential for recruitment is very low. Please remember to introduce the study at a suitable time preferably with a **FORM A** consent to contact.

Thanks again for all your work and continued support.

In this issue:

- SNOD NEWS
- Wales Kidney Research Unit
- Race Equality First
- Pet Idol 2016
- Llanelli Multicultural Network

Follow us

http://organ-donation-project.bangor.ac.uk/

OrganDonation_

organdonationproject

****

Contact us

Email: organdonationproject@bangor.ac.uk

Tel: 01248 382342

**Study Progress Report** Quick look table	NORTH	SOUTH
FORM As Consent to Contact	8	36
FORM Bs SNOD Questionnaire	25	43
FORM Cs Family Questionnaire	2	11

Interviews completed	28
Interviews in progress	16

The **Wales Kidney Research Unit** will be opening it's doors to the public on: **Wed - 12th of October 1.00-5.00pm** at the Nephrology Division of Infection and Immunity Cardiff University School of Medicine.

Please feel free to pop in to learn more about what the unit does, tour the labs, show your support, highlight anything that is important to you, or just to learn more about Kidney research in Wales. The Organ Donation Project is one of the many projects run out of the Unit. There will be refreshments and games! **Click here** for more details.

Thanks to **Race Equality First** for their invitation to present on Radio Cardiff this week.

Study Researcher Officer Leah took the 2.30 slot to talk about the Organ Donation Project and promote organ donation week within BAME Communities. Read more about Radio Cardiff here and the Race Equality First show:

http://radiocardiff.org/our-shows/race-equality-first-show/

Wales Pet Idol 2016

A huge **congratulations** to Specialist Nurse in Organ Donation "Charlotte" and her bilingual dog "Blodyn" who won the **Wales Pet Idol 2016** competition last week.

The prize was a year's supply of pet food! Read more about Blodyn and her bilingual abilities here:

http://www.walesonline.co.uk/news/local-news/bilingual-blodyn-crowned-winner-pet-11855777

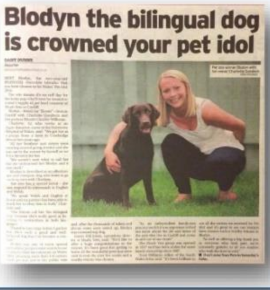

Blodyn the bilingual dog is crowned your pet idol

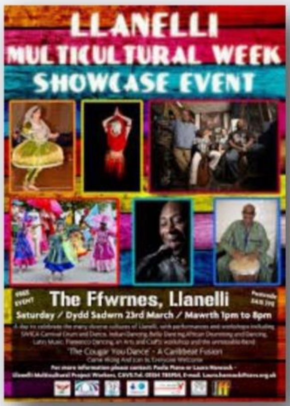

Llanelli Multicultural Network

Many thanks to Ann Evans - Chair of Llanelli Multi Cultural Network for inviting **Study Research Officer Leah** to come and speak at one of their drop in sessions last week. The network hosts a diverse range of activities for the Llanelli community and is supported by local volunteers. Leah got to talk about the Organ Donation at a break during an English lesson and to learn more about how peoples whose second language is English have understood the changes to the law in the Wales as well as have a general discussions about organ donation. Looking forward to attending more events and meeting more of the community in the future. You can hear about upcoming events as well as show your support here: https://www.facebook.com/LMCN-133503916744820/about/?entry_point=page_nav_about_item&tab=page_info
