# Supplementary material for: Designing a co‐productive study to overcome known methodological challenges in organ donation research with bereaved family members
Source: Health Expect. 2019 May 6;22(4):824–35. doi: 10.1111/hex.12894 (PMC6737840; doi:10.1111/hex.12894)

Supplemental file 4. Examples of co-productive communications with study partners.

The examples of co-production and on-going study communication.

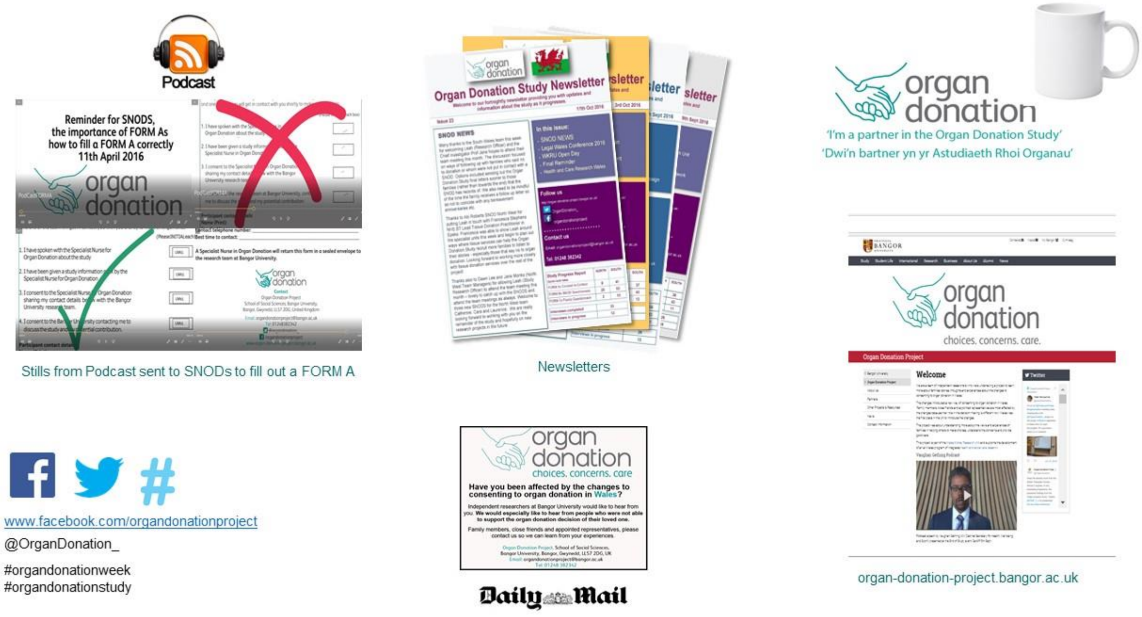

Supplement: Supplementary file 4 [file HEX-22-824-s004.pdf]
