# Supplementary material for: Designing a co‐productive study to overcome known methodological challenges in organ donation research with bereaved family members
Source: Health Expect. 2019 May 6;22(4):824–35. doi: 10.1111/hex.12894 (PMC6737840; doi:10.1111/hex.12894)
Supplement: Supplementary file 5 [file HEX-22-824-s005.pdf]

Supplementary file 5. Framework for ethical decision making.

Table 1. Framework for ethical decision-making.

Ethical considerations	Practical strategies
Participant identification and recruitment	
Access, confidentiality	Formally obtain the support of a key person to undertake the role of identifying potential participants and disseminating pre-prepared recruitment packs on behalf of the research team.
Regard	Recruit potential participants in a serial manner, for example, send out a maximum of five recruitment packs at any one time so that participants are not kept waiting for long periods before the research interview.
Respect, relevance	Consider participant inclusion criteria of bereaved no less than 3 months and no more than 12 months at the time of recruitment to the study.
Compassion	Include a covering letter that introduces the study in a personalised way by taking familiarity into consideration.
Informed choice	Provide clear written and web-based information about the researchers and the study. Include an invitation to contact the researcher. Demonstrate timely responsiveness to any potential questions or queries.
Non-coercion	Provide a minimum of 10 days for participants to decide about joining the study.
The research interview	
Choice, respect	Agree a convenient date, time and venue for the research interview. Avoid dates that coincide with any significant family events or anniversaries.
Safety	Implement a study site policy for researchers working alone in advance of the interview encounter.
Safety, support	Competent researcher with experience of conducting sensitive research interviews and supporting the bereaved.
Choice, privacy	Provide the option of an interview face to face or remotely, for example, via telephone.
Informed consent	Provide an overview of the study and present opportunity for participants to ask questions. Explain how the interview will proceed. Obtain written agreement to audio-record the interview and to use anonymous quotes in any presentation of the research. Provide participants with a copy of the signed consent form to keep.
Support	Discuss and agree avenues of post-interview support prior to the interview commencing.
Support	Observe/listen for signs of distress during the interview. Discuss the option of pausing the recording or stopping the interview. Plan a natural break for refreshments.
Confidentiality, anonymity	Ensure audio-recordings and transcripts are securely stored and electronic data are password protected. Assign a study code at the point of transcription.
Post-interview follow-up care	
Support	Arrange a convenient time to telephone the participant (normally in 24–48 h) to check on any issues the interview may have raised and to answer any questions.
Support	Compile information about local support organisations. Offer this to participants if they consider it helpful and/or direct them to appropriate professionals to discuss any issues of concern.
Support	Establish if participants wish their general practitioner (GP) to be informed about their participation in the study and obtain written consent to proceed. Provide GP with information about the study at the time of notification.
Appreciation	Send participants a personal thank-you letter and offer an executive summary of the research findings.
Involvement	Provide participants with an opportunity to evaluate their experience of participating in bereavement research.
Support	Determine support for the researcher from an individual with whom they feel comfortable and who is suitably qualified to provide support. Plan a debriefing session after each interview encounter. Utilise reflexive notes to guide the discussion.

Table 1. Framework for ethical decision-making.

Ethical considerations	Practical strategies
Participant identification and recruitment	
Access, confidentiality	Formally obtain the support of a key person to undertake the role of identifying potential participants and disseminating pre-prepared recruitment packs on behalf of the research team.
Regard	Recruit potential participants in a serial manner, for example, send out a maximum of five recruitment packs at any one time so that participants are not kept waiting for long periods before the research interview.
Respect, relevance	Consider participant inclusion criteria of bereaved no less than 3 months and no more than 12 months at the time of recruitment to the study.
Compassion	Include a covering letter that introduces the study in a personalised way by taking familiarity into consideration.
Informed choice	Provide clear written and web-based information about the researchers and the study. Include an invitation to contact the researcher. Demonstrate timely responsiveness to any potential questions or queries.
Non-coercion	Provide a minimum of 10 days for participants to decide about joining the study.
The research interview	
Choice, respect	Agree a convenient date, time and venue for the research interview. Avoid dates that coincide with any significant family events or anniversaries.
Safety	Implement a study site policy for researchers working alone in advance of the interview encounter.
Safety, support	Competent researcher with experience of conducting sensitive research interviews and supporting the bereaved.
Choice, privacy	Provide the option of an interview face to face or remotely, for example, via telephone.
Informed consent	Provide an overview of the study and present opportunity for participants to ask questions. Explain how the interview will proceed. Obtain written agreement to audio-record the interview and to use anonymous quotes in any presentation of the research. Provide participants with a copy of the signed consent form to keep.
Support	Discuss and agree avenues of post-interview support prior to the interview commencing.
Support	Observe/listen for signs of distress during the interview. Discuss the option of pausing the recording or stopping the interview. Plan a natural break for refreshments.
Confidentiality, anonymity	Ensure audio-recordings and transcripts are securely stored and electronic data are password protected. Assign a study code at the point of transcription.
Post-interview follow-up care	
Support	Arrange a convenient time to telephone the participant (normally in 24–48 h) to check on any issues the interview may have raised and to answer any questions.
Support	Compile information about local support organisations. Offer this to participants if they consider it helpful and/or direct them to appropriate professionals to discuss any issues of concern.
Support	Establish if participants wish their general practitioner (GP) to be informed about their participation in the study and obtain written consent to proceed. Provide GP with information about the study at the time of notification.
Appreciation	Send participants a personal thank-you letter and offer an executive summary of the research findings.
Involvement	Provide participants with an opportunity to evaluate their experience of participating in bereavement research.
Support	Determine support for the researcher from an individual with whom they feel comfortable and who is suitably qualified to provide support. Plan a debriefing session after each interview encounter. Utilise reflexive notes to guide the discussion.
