# Supplementary material for: Designing a co‐productive study to overcome known methodological challenges in organ donation research with bereaved family members
Source: Health Expect. 2019 May 6;22(4):824–35. doi: 10.1111/hex.12894 (PMC6737840; doi:10.1111/hex.12894)
Supplement: Supplementary file 6 [file HEX-22-824-s006.pdf]

Supplemental file 6. Distress Protocol.

Distress Protocol

During instances of bereaved participants becoming distressed during the interview process, the subsequent protocol will be followed.

Identifying Distress

The interviewer will be mindful of signs of distress in the participants throughout the interviews. Signs of distress to look out for will include:

- Exhibition of behaviours that indicate that the discussion has become too upsetting for them, including crying and an inability to continue for example.
- The participant verbally communicating that they are experiencing distress during the interview.

Response Stage 1

- The interview will be stopped.
- The participant will be offered a break, have a drink of water/ tea etc.
- The participant will then be asked if they would like to continue the interview or if they would prefer to discontinue. Should they wish to go on, the interview will resume.

Response Stage 2

If the participant elects to discontinue the interview,

- The interview will not continue.
- The interviewer will signpost where support can be obtained such as from the SNOD or the bereavement support service at the hospital where their relative/friend died, or from their GP.
- The participants will also be reminded again of the contact details for Cruise Bereavement Care,' an organisation which provides support for bereaved people.

Response Stage 3

- At a later date, the interviewer will follow up with the participant with a courtesy call (with the participant's consent). If the participant feels strongly that they would still like to have their views and experiences heard – the interviewer will go through options (wait a while before rearranging, explore other methods rather than face to face etc.
