# Supplementary material for: Designing a co‐productive study to overcome known methodological challenges in organ donation research with bereaved family members
Source: Health Expect. 2019 May 6;22(4):824–35. doi: 10.1111/hex.12894 (PMC6737840; doi:10.1111/hex.12894)
Supplement: Supplementary file 7 [file HEX-22-824-s007.pdf]

Supplemental file 7. End of study celebration event.

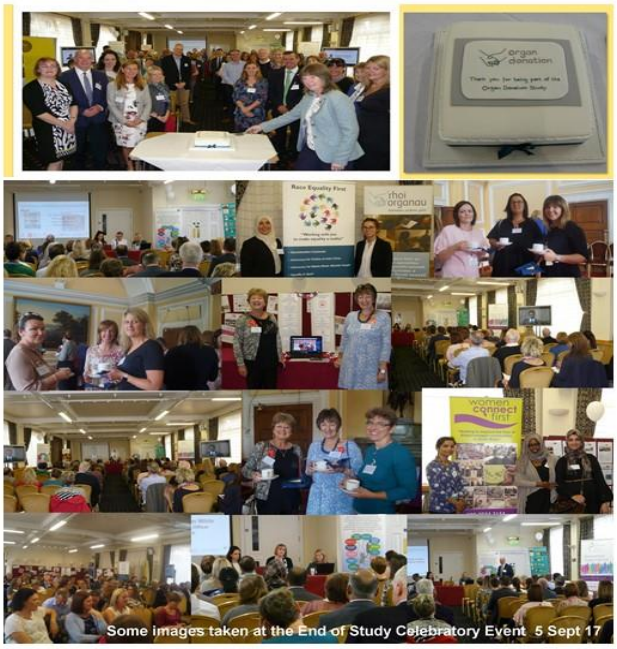

End of study celebration event. Chief Nursing officer responded to findings, Janette Bourne presented on co-production, and Phil Walton closed the Event.
